# Population‐based differences in the outcome and presentation of lung cancer patients based upon racial, histologic, and economic factors in all lung patients and those with metastatic disease

**DOI:** 10.1002/cam4.1430

**Published:** 2018-03-13

**Authors:** John Michael Varlotto, Richard Voland, Kerrie McKie, John C. Flickinger, Malcolm M. DeCamp, Debra Maddox, Paul Rava, Thomas J. Fitzgerald, Geoffrey Graeber, Negar Rassaei, Paulo Oliveira, Suhail Ali, Chandra Belani, Jonathan Glanzman, Heather A. Wakelee, Manali Patel, Jennifer Baima, Jianying Zhang, William Walsh

**Affiliations:** ^1^ Department of Radiation Oncology University of Massachusetts Medical Center Worcester Massachusetts; ^2^ University of Massachusetts Medical School Worcester Massachusetts; ^3^ School of Nursing University of Wisconsin Madison Wisconsin; ^4^ Department of Radiation Oncology University of Pittsburgh Medical Center Pittsburgh Pennsylvania; ^5^ Division of Thoracic Surgery Feinberg School of Medicine Northwestern University Chicago Illinois; ^6^ Department of Medical Oncology University of Massachusetts Medical Center Worcester Massachusetts; ^7^ Division of Thoracic Surgery University of Massachusetts Medical Center Worcester Massachusetts; ^8^ Department of Pathology Penn State Hershey Medical Center Hershey Pennsylvania; ^9^ Division of Pulmonary, University of Massachusetts Medical Center, Allergy and Critical Care Medicine Worcester Massachusetts; ^10^ Penn State Hershey Cancer Institute Hershey Pennsylvania; ^11^ Division of Medical Oncology Stanford University Palo Alto California; ^12^ Department of Orthopedics and Rehabilitation University of Massachusetts Medical Center Worcester Massachusetts; ^13^ Department of Quantitative Sciences University of Massachusetts Medical School Worcester Massachusetts

**Keywords:** Disparities, insurance, lung cancer, marriage, metastatic lung cancer, outcomes, race, socio‐economic factors

## Abstract

To investigate the interrelation between economic, marital, and known histopathologic/therapeutic prognostic factors in presentation and survival of patients with lung cancer in nine different ethnic groups. A retrospective review of the SEER database was conducted through the years 2007–2012. Population differences were assessed via chi‐square testing. Multivariable analyses (MVA) were used to detect overall survival (OS) differences in the total population (TP,* N* = 153,027) and for those patients presenting with Stage IV (*N* = 70,968). Compared to Whites, Blacks were more likely to present with younger age, male sex, lower income, no insurance, single/widowed partnership, less squamous cell carcinomas, and advanced stage; and experience less definitive surgery, lower OS, and lung cancer‐specific (LCSS) survival. White Hispanics presented with younger age, higher income**,** lower rates of insurance, single/widowed partnership status, advanced stage, more adenocarcinomas, and lower rates of definitive surgery, but no difference in OS and LCSS than Whites. In the TP and Stage IV populations, MVAs revealed that OS was better or equivalent to Whites for all other ethnic groups and was positively associated with insurance, marriage, and higher income. Blacks presented with more advanced disease and were more likely to succumb to lung cancer, but when adjusted for prognostic factors, they had a better OS in the TP compared to Whites. Disparities in income**,** marital status, and insurance rather than race affect OS of patients with lung cancer. Because of their presentation with advanced disease, Black and Hispanics are likely to have increased benefit from lung cancer screening.

## Introduction

In the United States, lung cancer occurs in approximately 225,000 patients and is associated with over 160,000 deaths annually 1. However, despite the prevalence of this malignancy, the influence and/or interrelation between economic and insurance factors as well as ethnicity have been poorly studied. One recent study did investigate racial/ethnic differences in lung cancer incidence and mortality in women, but found no differences in fully adjusted models 2. Additionally, another report demonstrated lung cancer rates have dropped faster in Black women than the rates in Whites since the 1990s 3. Using the Christiana Care Tumor Registry (CCTR) in Delaware, disparities in survival were related to lower socioeconomic status and having Medicaid insurance, but there were no such differences related to race or sex 4. A National Cancer Database project 5 found income and race (White, Black, and Asian) were not related to survival, but patients with Medicaid or who were uninsured had worse outcomes 5.

The purpose of our analysis was to study whether marital status, household income, insurance type, and ethnicity play a role in the presentation and prognosis of all patients with NSCLC and those presenting with metastatic disease in the United States. We feel this investigation is unique because we investigate the racial groups in terms of their presenting economic, histopathologic, and marital status and assess whether racial differences account for differing prognoses.

## Materials and Methods

### Data source/cohort selection

The “SEER‐18” database was available since the year 2000 6 and covers approximately 28% of the American population 6. The years 2007–2012 were queried to identify patients with microscopically confirmed NSCLC as their first primary tumor.

Outcome and presenting characteristics were examined for all patients (153,027) and patients with metastatic disease (70,968) for whom sufficient information was collected to assess the outcomes in relation to patient, economic, histopathologic, and insurance variables.

### Outcome variables and other covariates

The main purpose of our analysis was to examine whether there were differences in presenting characteristics and outcome in nine different ethnic groups by examining marital status, household income, and insurance type in addition to established histopathologic (tumor location, size, differentiation, stage, and histology), treatment factors (radiation and definitive surgical procedure), and patients factors (age, gender, marital status, presenting year, and SEER registry site). The patients with lung cancer were split into nine different ethnic groups as follows: White non‐Hispanic (White), Black, White Hispanic (Hispanic), American Indian/Alaskan native (AI/AN), Chinese, Japanese, South Asian (Asian Indian and Pakistani), other Asians (OA, Filipino, Thai, Vietnamese, Korean, Kampuchean, Laotian, and Hmong), and other races (OR, Chamorran, Fiji Islander, Guamanian, Hawaiian, Melanesian, Micronesian, New Guinean, Pacific Islander, Polynesian, Samoan, Tahitian, Tongan, unknown, and others) in both the TP and Stage IV populations. The number of Black Hispanic patients was scant in both the TP (1.0%) and the Stage IV groups (0.7%), thus precluding the possibility of considering a separate patient category, and thus, Black Hispanic patients are included in the Black category, similar to a past study 7. Throughout this manuscript, the term population(s) will refer to total population of patients with lung cancer and those with Stage IV disease, while group(s) will refer to the nine different ethnicities.

At the time of our analysis, SEER did not contain information regarding whether systemic therapy was given, nor does SEER contain information regarding the systemic agents that were used. SEER does contain information including the following: year of diagnosis (1975–2014), sex, patient age (1–84 and 85+), SEER registry location, median household income, insurance, marital status, origin recode (Hispanic, non‐Hispanic), race/ethnicity, tumor location, primary site, sequence number, grade, laterality, tumor size, tumor extension, number of nodes examined, number of nodes positive, TNM stages, Mets at diagnosis, type of surgery, cause of death, vital status, and survival months.

### Statistical analysis

Chi‐square and *t*‐tests compared differences between the ethnic groups with respect to treatment and patient/tumor characteristics. Cox proportional hazards models (Therneau, Grambsch) 8 were used to calculate adjusted hazard ratios with their 95% confidence intervals and to show how treatment and other covariates were related to overall survival OS and LCSS. Medicare eligibility was controlled through use of two strata for age at diagnosis (≥65 years old vs. <65 years) because individual cases will change when they enroll in Medicare.

## Results

Median follow‐up time was calculated by the methods of Schemper and Smith in which death becomes a censored follow‐up time and was noted to be 35 and 31 months in the TP and Stage IV groups, respectively 9.

Complete demographic and histologic details of the TP (153,027) and Stage IV (70,968) populations can be seen in Table 1. Median age in the TP and Stage IV are 68.0 and 67.0, respectively. There was a male predominance to both populations (54.1%‐ TP, and 55.8%‐ Stage IV). The three largest ethnic groups in the TP and Stage IV population were White, Black, and Hispanic and were 74.4%, 12.3%, and 5.7%; and 72.3%, 13.2%, and 6.3%, respectively. A similar proportion of the Stage IV (31.7%) and TP (32.3%) patients presented with a low median family income (<$50,000). The majority were married with 51.6% and 51.2% in the TP and Stage IV, respectively. 82.3% and 80.1% of TP and Stage IV patients were insured. Adenocarcinoma was the predominant histology in both populations (52.6%, TP; and 55.5%, Stage IV).

**Table 1:** Demographic characteristics of both the TP and Stage IV patients

	All Patients (TP) *N* = 153,027	Stage IV patient *N* = 70,968
Age—year, median	68.0	67.0
Sex		
Female	70,212 (45.9%)	31,353 (44.2%)
Male	82,815 (54.1%)	39,615 (55.8%)
Race		
White Hispanic	8579 (5.6%)	4441 (6.3%)
White non‐Hispanic	114,013 (74.5%)	51,296 (72.3%)
Black	18,852 (12.3%)	9360 (13.2%)
Chinese	2413 (1.6%)	1261 (1.8%)
Japanese	1229 (0.80%)	567 (0.80%)
South Asian	451 (0.29%)	238 (0.34%)
Other Asians	4831 (3.2%)	2544 (3.6%)
Other Races	1957 (1.3%)	943 (1.3%)
American Indian/Alaskan Native	702 (0.46%)	318 (0.45%)
SEER registry		
Alaska Natives	199 (0.13%)	76 (0.11%)
Atlanta	4629 (3.0%)	2226 (3.1%)
California excl SF/SJM/LA	30,007 (19.6%)	14,304 (20.2%)
Connecticut	8088 (5.30%)	3639 (5.1%)
Detroit	9852 (6.4%)	4632 (6.5%)
Greater Georgia	14,260 (9.3%)	6380 (9.0%)
Hawaii	2375 (1.6%)	1159 (1.6%)
Iowa	6805 (4.4%)	3205 (4.5%)
Kentucky	13,916 (9.1%)	5980 (8.4%)
Los Angeles	11,437 (7.5%)	5789 (8.2%)
Louisiana	10,783 (7.0%)	4767 (6.7%)
New Jersey	17,451 (11.4%)	7796 (11.0%)
New Mexico	2610 (1.7%)	1215 (1.7%)
Rural Georgia	353 (0.23%)	140 (0.20%)
San Francisco–Oakland	7081 (4.6%)	3469 (4.9%)
San Jose–Monterey	3203 (2.1%)	1635 (2.3%)
Seattle	8271 (5.4%)	3726 (5.3%)
Utah	1707 (1.1%)	830 (1.2%)
Income		
<$50,000	49,407 (32.3%)	22,524 (31.7%)
$50,000–74,000	81,027 (52.9%)	37,933 (53.5%)
≥75,000	22,593 (14.8%)	10,511 (14.8%)
Marital status		
Divorced	18,851 (12.3%)	8815 (12.4%)
Married	78,957 (51.6%)	36,349 (51.2%)
Separated	1785 (1.2%)	895 (1.3%)
Single	21,126 (13.8%)	10,872 (15.3%)
Unknown	6032 (3.9%)	2866 (4.0%)
Domestic Partner	126 (0.082%)	60 (0.084%)
Widowed	26,150 (17.1%)	11,111 (15.7%)
Tumor stage		
Unknown	3174 (2.0%)	0
I	34,255 (22.3%)	0
II	7825 (5.1%)	0
III	39,979 (26.1%)	0
IV	70,968 (46.3%)	70,968 (100.0%)
Insurance		
Insured	125,876 (82.3%)	56,859 (80.1%)
Medicaid	20,741 (13.6%)	10,324 (14.5%)
Uninsured	5272 (3.4%)	3145 (4.4%)
Unknown	1138 (0.74%)	640 (0.96%)
Lateral location		
Bronchus, Left	2427 (1.6%)	1311 (1.8%)
Bronchus, Right	2427 (2.2%)	1875 (2.6%)
Bronchus, Unknown	168 (0.11%)	103 (0.15%)
Left Lower	17,384 (11.4%)	7266 (10.2%)
Left Upper	35,216 (23.0%)	14,827 (20.9%)
Left NOS	4733 (3.1%)	2993 (4.2%)
Lung, NOS	6207 (4.1%)	5382 (7.6%)
Left Overlapping	507 (0.32%)	249 (0.35%)
Right Lower	22,457 (14.7%)	9476 (13.4%)
Right Middle	6376 (4.2%)	2848 (4.0%)
Right Upper	45,478 (29.7%)	19,134 (27.0%)
Right NOS	7460 (4.9%)	4921 (6.9%)
Right Overlapping	1268 (0.83%)	583 (0.82%)
Histology—No. (%)		
Adenocarcinoma	80,499 (52.6%)	39,415 (55.5%)
Squamous Cell Ca	41,573 (27.2%)	14,239 (20.0%)
Non‐small‐cell Ca, NOS	22,886 (15.0%)	13,603 (19.2%)
Large Cell Ca	4519 (3.0%)	2301 (3.2%)
Adenosquamous Cell Ca	2442 (1.6%)	921 (1.3%)
Others	1108 (0.72%)	489 (0.69%)
Grade		
Well, I	8365 (5.4%)	1795 (2.5%)
Moderately, II	30,010 (19.6%)	7974 (11.2%)
Poorly, III	45,364 (29.6%)	19,445 (27.3%)
Undifferentiated, IV	2497 (1.6%)	1142 (1.6%)
Unknown	66,791 (43.8%)	40,612 (57.2%)
Definitive surgical procedure	39,105 (25.6%)	
Radiation given	64,552 (41.8%)	33,689 (47.5%)
Year of diagnosis		
2007	25,396 (16.6%)	11,589 (16.3%)
2008	25,529 (16.7%)	11,572 (16.3%)
2009	25,650 (16.8%)	11,848 (16.7%)
2010	25,631 (16.7%)	12,135 (17.1%)
2011	25,459 (16.6%)	11,807 (16.6%)
2012	25,362 (16.6%)	12,017 (16.9%)

**Figure 1:**
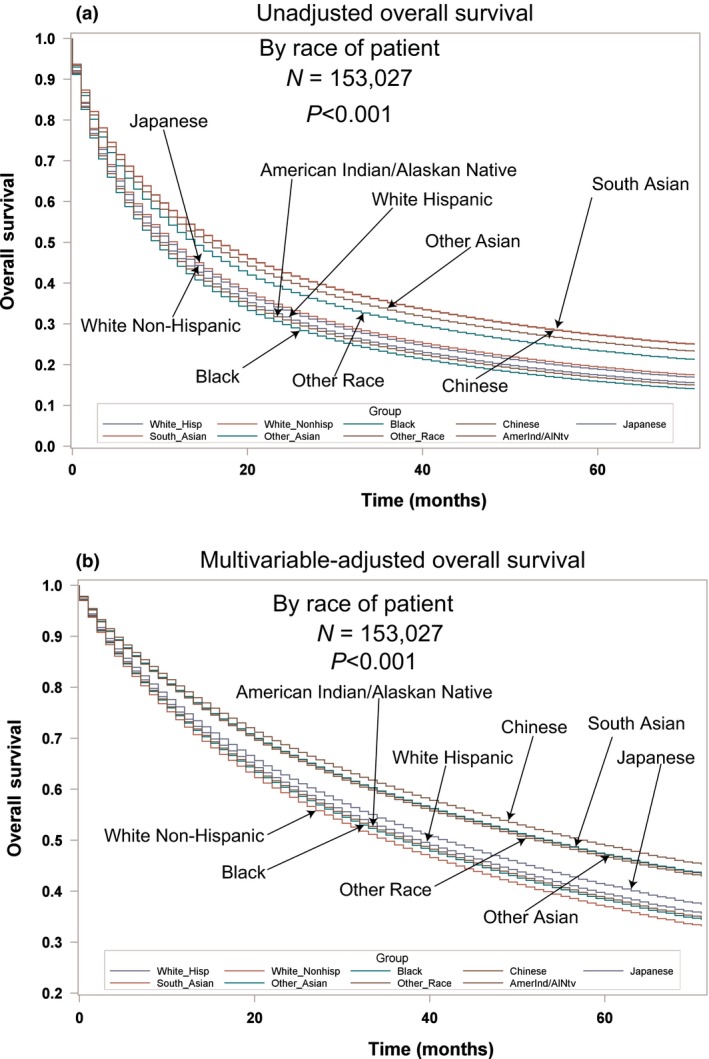
(A) Unadjusted overall survival by ethnic group in the total population. (B) Multivariable‐adjusted overall survival by ethnic group in the total population.

Table S1 supplemental contains the unadjusted demographic, histologic, and treatment details in the TP and used Whites as the reference group. Blacks were presented with younger age, more males, lower median household income, more uninsured, higher stages, lower percentage of squamous cell carcinomas, lower rates of definitive surgery, and lower OS/LCSS. Hispanics were presented at a younger age, higher median household income, more uninsured, higher percentage of metastatic disease, higher percentage of adenocarcinomas, and lower rates of definitive surgery, but had a similar OS/LCSS. The Japanese were presented with a highest mean age (72.8), the only female predominance (51.2%), and the highest rates of insurance (96.4%), but there were a similar OS and LCSS compared to Whites. Whites were presented with the higher percentage of Stage I disease (23.4%) than all except for the South Asian and AI/AN. South Asians were presented with the highest percentage of metastatic disease at 52.8%. The Chinese were presented with the highest percentage of adenocarcinomas (69.4%), while AI/AN were presented with the highest percentage of squamous cell carcinomas (30.8%). Whites had significantly higher rates of definitive surgical procedures except for the Chinese, Japanese, and South Asians. As compared to the White population, OS and LCSS were significantly greater in the Chinese, South Asians, other Asians, and other racial groups. Blacks had a lower OS and LCSS. Unadjusted OS by ethnic group can be found in Figure 1A.

**Figure 2:**
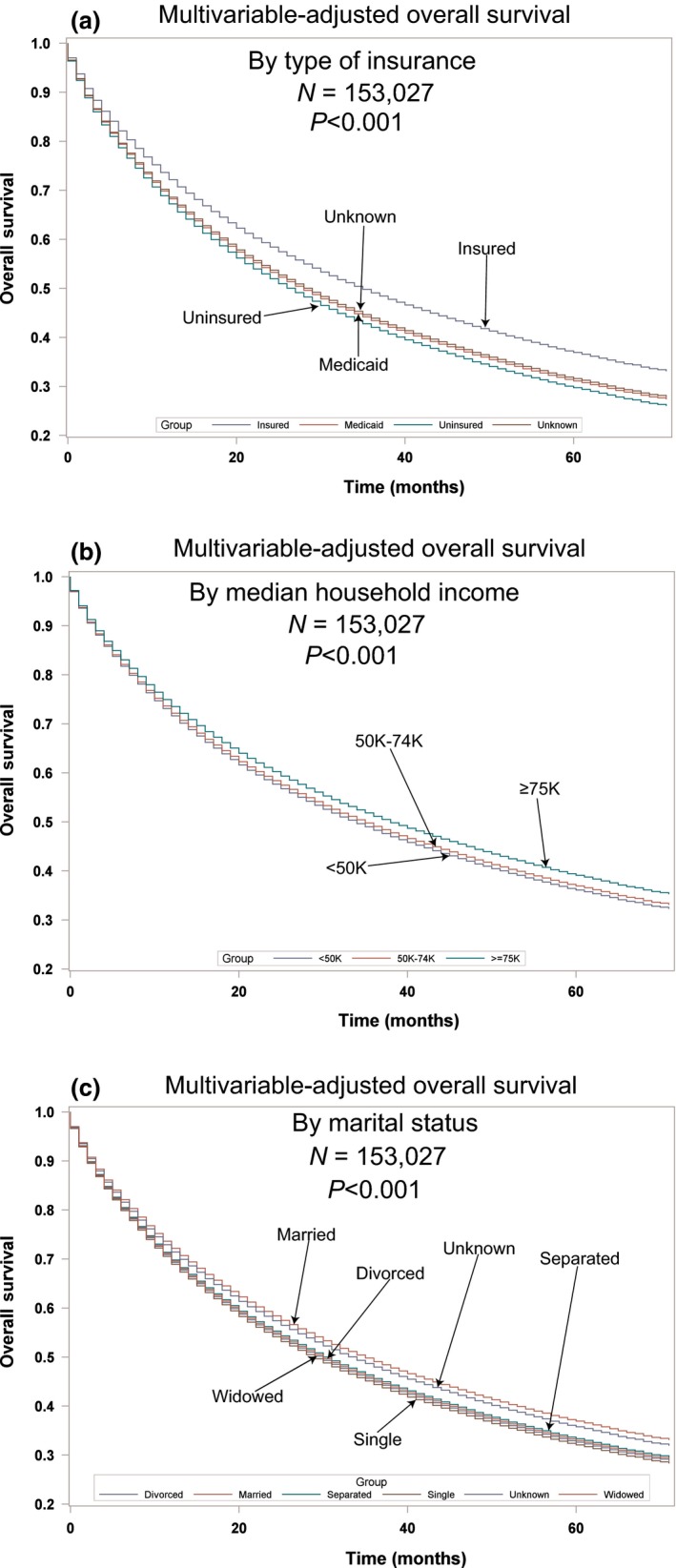
Multivariable‐adjusted overall survival in the total population. (A) by insurance; (B) by income; (C) by marital status.

Multivariable analyses for OS and MVA‐adjusted OS in the TP can be seen in Table 2 and Figure 1B. Advancing age (*P* < 0.0001, HR = 1.185) and male sex (*P* < 0.0001, HR = 1.245) were associated with worse OS. Whites had a lower OS than all races (HR = 0.705–0.977) except for AI/AN who had a similar OS (*P* = 0.4890, HR = 0.963). OS was lower for lower (*P* = 0.0097, HR = 1.024) and better for higher median household incomes (*P* < 0.001, HR = 0.936). Insured patients had a better OS than the uninsured, those on Medicaid and those with unknown insurance (all *P* < 0.0001, HR = 1.197–1.246). Married patients had a better OS than separated, single, widowed, and unknown (all *P* ≤ 0.0004, HR = 1.062–1.166). As compared to Stage I, Stages II‐IV had a worse OS with increasing HRs with stage (all *P* < 0.0001, HR = 1.622–3.290). The lower lobes and mainstem bronchi locations were associated with worse OS. All histologies had a worse OS (all *P* < 0.0001, HR = 1.113–1.536) than adenocarcinomas. Compared to well‐differentiated tumors, other tumor grades had worse OS (all *P* < 0.0001, HR = 1.372–1.731). Patients who received radiation (*P* < 0.0001, HR = 0.759) or definitive surgery (*P* < 0.0001, HR = 0.331) had a better OS. OS by insurance, income, and marital status for TP can be seen in Figure 2A–C.

**Table 2:** Multivariate analysis for OS in TP, *N* = 153,207

All (*N* = 153,207)	*P*‐value	Hazard ratio
Age—year	1.185	<0.0001
Sex		
Female	–	1.0
Male	<0.0001	1.245
Race		
White non‐Hispanic	–	1.0
White Hispanic	<0.0001	0.937
Black	0.0205	0.977
Chinese	<0.0001	0.705
Japanese	0.0061	0.903
South Asian	<0.0001	0.733
Other Asians	<0.0001	0.762
Other Races	<0.0001	0.792
American Indian/Alaskan Native	0.4890	0.963
SEER Registry		
Alaska Natives	0.3002	1.111
Atlanta	0.1724	1.032
California excl SF/SJM/LA	0.0003	1.060
Connecticut	–	1.0
Detroit	0.3908	1.017
Greater Georgia	<0.0001	1.095
Hawaii	<0.0001	1.149
Iowa	0.0002	1.081
Kentucky	<0.0001	1.135
Los Angeles	0.3514	0.983
Louisiana	<0.0001	1.141
New Jersey	0.0117	1.044
New Mexico	0.6428	1.014
Rural Georgia	0.9719	0.998
San Francisco–Oakland	0.0325	1.046
San Jose–Monterey	0.1455	1.040
Seattle	0.2381	1.024
Utah	0.0011	1.114
Income		
<$50,000	0.0097	1.024
$50,000–74,000	–	1.0
≥75,000	<0.0001	0.936
Insurance		
Insured		1.0
Medicaid	<0.0001	1.200
Uninsured	<0.0001	1.246
Unknown	<0.0001	1.197
Marital status		
Married	–	1.0
Divorced	<0.0001	1.144
Separated	0.0001	1.120
Single	<0.0001	1.166
Unknown	0.0004	1.062
Domestic Partner	0.3785	1.124
Widowed	<0.0001	1.147
Tumor stage		
I	–	1.0
II	<0.0001	1.622
III	<0.0001	1.994
IV	<0.0001	3.290
Lateral location		
Right upper	–	1.0
Bronchus, Left	<0.0001	1.232
Bronchus, Right	<0.0001	1.297
Bronchus, Unknown	0.0169	1.226
Left Lower	<0.0001	1.062
Left Upper	0.0874	1.015
Left NOS	<0.0001	1.228
Lung, NOS	<0.0001	1.211
Left Overlapping	0.0077	1.152
Right Lower	<0.0001	1.083
Right Middle	0.2052	1.022
Right NOS	<0.0001	1.253
Right Overlapping	<0.0001	1.313
Histology—No. (%)		
Adenocarcinoma	–	1.0
Adenosquamous	<0.0001	1.196
Large Cell	<0.0001	1.176
Nonsmall Cell	<0.0001	1.149
Others	<0.0001	1.536
Squamous	<0.0001	1.113
Grade		
Well, I	–	1.0
Moderately, II	<0.0001	1.372
Poorly, III	<0.0001	1.629
Undifferentiated, IV	<0.0001	1.731
Unknown	<0.0001	1.537
Definitive Surgical Procedure	<0.0001	0.331
Radiation	<0.0001	0.759
Year of diagnosis		
2007	–	1.0
2008	0.0664	0.982
2009	0.0002	0.963
2010	<0.0001	0.956
2011	<0.0001	0.914
2012	<0.0001	0.891

Multivariable analyses for OS for the Stage IV population can be seen in Table 3. Age (*P* < 0.0001, HR = 1.017) and male sex (*P* < 0.0001, HR = 1.233) were associated with worse OS. All races had better OS than Whites (HR = 0.709–0.898) except for AI/AN and Blacks who had a similar OS. OS decreased for lower incomes (*P* = 0.0484, HR = 1.025) and increased for higher incomes (*P* < 0.001, HR = 0.934). Insured patients had a better OS than uninsured and those with Medicaid and unknown insurance (all *P* < 0.0001, HR = 1.195–1.277). Married patients or those with a domestic partner had better OS than those not living in a stable partner situation (divorced, *P* < 0.0001, HR = 1.154; widowed, *P* < 0.0001, HR = 1.149; separated, *P* = 0.0009, HR = 1.134; and unknown, *P* = 0.0023, HR = 1.069). Involvement of mainstem bronchi and right lower lobe was deleterious for OS. All other histologies were associated with a worse OS compared to adenosquamous cell carcinoma or adenocarcinoma (all *P* < 0.0001, HR = 1.107–1.482). All tumors differentiation compared to well‐differentiated had significantly worse OS (all *P* < 0.0001, HRs 1.258–1.702). Palliative radiation significantly improved OS (*P* < 0.0001, HRs 0.896). Starting in the year 2010, OS started to significantly improve with progressively lower HRs each year.

**Table 3:** Multivariate Analysis for OS in Stage IV population, *N* = 70,968

All (*n* = 70,968)	*P*‐value	Hazard ratio
Age—year	<0.0001	1.017
Sex		
Female	–	1.0
Male	<0.0001	1.233
Race		
White non‐Hispanic	–	1.0
White Hispanic	<0.0001	0.924
Black	0.1704	0.982
Chinese	<0.0001	0.709
Japanese	0.0281	0.898
South Asian	<0.0001	0.729
Other Asians	<0.0001	0.776
Other Races	<0.0001	0.800
American Indian/Alaskan Native	0.9669	0.997
SEER Registry		
Alaska Natives	0.3708	1.136
Atlanta	0.6764	0.987
California excl SF/SJM/LA	0.2900	1.022
Connecticut	–	1.0
Detroit	0.7774	1.007
Greater Georgia	0.0971	1.042
Hawaii	0.0229	1.097
Iowa	0.0442	1.057
Kentucky	0.0487	1.052
Los Angeles	0.0346	0.950
Louisiana	0.0044	1.078
New Jersey	0.9390	0.998
New Mexico	0.9163	1.004
Rural Georgia	0.9052	1.011
San Francisco–Oakland	0.5813	1.015
San Jose–Monterey	0.4499	0.975
Seattle	0.9550	1.001
Utah	0.6367	1.020
Income		
<$50,000	0.0484	1.025
$50,000–74,000	–	1.0
≥75,000	<0.0001	0.934
Insurance		
Insured	–	1.0
Medicaid	<0.0001	1.195
Uninsured	<0.0001	1.273
Unknown	<0.0001	1.277
Marital status		
Married	–	1.0
Divorced	<0.0001	1.154
Separated	0.0009	1.134
Single	<0.0001	1.167
Unknown	0.0023	1.069
Domestic Partner	0.1157	1.283
Widowed	<0.0001	1.149
Lateral location		
Right upper	–	1.0
Bronchus, Left	<0.0001	1.210
Bronchus, Right	<0.0001	1.330
Bronchus, Unknown	0.0104	1.308
Left Lower	0.0607	1.029
Left Upper	0.5952	1.006
Left NOS	<0.0001	1.181
Lung, NOS	<0.0001	1.214
Left Overlapping	0.0241	1.168
Right Lower	0.0037	1.041
Right Middle	0.4196	0.982
Right NOS	<0.0001	1.163
Right Overlapping	0.0002	1.186
Histology—No. (%)		
Adenocarcinoma	–	1.0
Adenosquamous	0.0831	1.067
Large Cell	<0.0001	1.174
Nonsmall Cell	<0.0001	1.186
Others	<0.0001	1.482
Squamous	<0.0001	1.107
Grade		
Well, I		
Moderately, II	<0.0001	1.258
Poorly, III	<0.0001	1.627
Undifferentiated, IV	<0.0001	1.702
Unknown	<0.0001	1.645
Radiation	<0.0001	0.896
Number of Nodes examined		
Year of Diagnosis		
2007	–	1.0
2008	0.4704	0.990
2009	0.0684	0.976
2010	0.0332	0.971
2011	<0.0001	0.937
2012	<0.0001	0.899

## Discussion

A major finding of our analysis is Blacks often present at a younger age, have worse prognostic characteristics, and a lower OS/LCSS than Whites. However, after multivariable adjustment, Blacks have a better OS in the TP and similar OS in the Stage IV patients as compared to Whites. Blacks present with many poor prognostic factors including the following: lower median household income, single/widowed partnership status, higher male predominance, more uninsured, higher stages, and lower rates of definitive surgery. However, Blacks did present at a younger age and have a lower percentage of squamous cell carcinomas, both of which are associated with a better prognosis. As insurance, presentation stage, and surgical eligibility can be altered, there is hope that outcomes for Blacks can be improved with better access to insurance and by increased CT screening 10. In comparison with Whites, Hispanics presented with a higher proportion of several risk factors associated with poor prognosis including more uninsured, a lower proportion of Stage I/II tumors, and lower rates of definitive surgery, but there was no detrimental effect on the unadjusted OS or LCSS in the TP. Furthermore, MVAs demonstrated OS was significantly better for Hispanics compared to Whites in both the TP and Stage IV populations. It should be noted that previous analysis demonstrated this preferential OS benefit associated with Hispanics may be limited to those who are foreign‐born as compared to those born in the United States 11. Because the East Asian populations are enriched for the EGFR mutation tumors 12, it is not surprising the Chinese, South Asian, other Asians, and Japanese had a better adjusted OS in the TP/Stage IV populations, although this analysis lacks details on the mutational status of tumors.

In both MVAs for OS in the TP and Stage IV populations, male sex, poorer tumor differentiation, higher tumor stage, and advanced age were shown to be poor prognostic features and have been well established 13, 14. Furthermore, palliative radiation therapy was found to be important for OS in the Stage IV population. Involvement of the mainstem bronchi and lower lobes was associated with worse OS. Although it can be hypothesized the involvement of the mainstem bronchi can contribute to an increased mortality due to postobstructive pneumonia and/or hypoxia, the survival decrement noted with the lower lobe locations may be due to a greater involvement of normal lung volumes. Because of the known ability of radiation to alleviate symptoms in Stage IV lung cancer 15, we feel the OS benefit noted with radiation in this study may be due to its palliation of central‐based obstructive masses.

Since 2010 (2009 in TP), a consistent improvement in OS was noted in both populations. Although our analysis is unable to identify reasons for this progressive improvement, we feel the reasons are multifactorial. We speculate better staging with frequent use of CT/PET scanning 16 is associated with better outcomes. However, the benefits in the Stage IV population may have also been due to the recognition of chemotherapeutic regimens based upon histology 17 and benefits of targeted therapeutic agents for EGFR mutations 18 and EML4‐ALK translocations 19. Unfortunately, SEER does not contain information regarding the mutations or systemic therapy.

In both patient populations, MVA demonstrated higher income was positively associated with OS. Lower socioeconomic status was previously shown to affect cancer mortality and to be associated with modifiable risk factors such as smoking, diet, BMI, and lower levels of physical activity 20. Unfortunately, these modifiable risk factors are not contained within SEER‐18, but information concerning insurance is available and is more strongly correlated with OS than income. Furthermore, cigarette smoking is noted to be more prevalent in lower socioeconomic groups 21 and could account for the lower OS associated with economic factors. The effects of not being insured have greater effect on OS not only in this population group, but in our companion article concerning surgical patients in these same ethnic groups. It is interesting to note patients with Medicaid have similar hazard ratios for adverse outcomes as compared to those without insurance. We hypothesize the poor outcomes noted in the Medicaid population may be due to the socioeconomic conditions of individuals who have this coverage or possibly due to provider differences. Similar poor outcomes of patients who are receiving Medicaid or who are uninsured have recently been reported in patients with testicular cancers, glioblastomas, and head and neck cancers 22, 23, 24. Nevertheless, hopefully, Medicaid expansion will provide better health outcomes for patients with cancer and has already been associated with increases in medication adherence, preventive care, and healthcare quality 25. In a database of 75 countries obtained from the World Bank and WHO (1990–2010), unemployment was associated with increased lung cancer mortality, but only in men 26. The effects of unemployment on cancer mortality appeared to be mitigated by universal health coverage. Our results suggest the type of insurance can affect the prognosis of patients with short expected survivals, that is, Stage IV. Although higher lung cancer mortality was recently noted in the mid‐South 27 and our analysis indicates there is worse OS in Kentucky and Louisiana in both patient populations, the effects of geography on poor prognosis in our study were not limited to those areas. Our results show married patients or those with a domestic partner have a significantly longer survival, even in metastatic disease. Although our results conflict with those of a past investigation 28 in patients with lung cancer, other investigators have noted unmarried patients with lung cancer had a greater incidence of depression, less social support, and a survival decrement 29.

SEER‐18 lacks many variables including smoking, diet, BMI, levels of physical activity, type of chemotherapeutic agents, radiation doses/volumes, surgical complications, medical office visits, and patient comorbidities. Therefore, our analysis cannot account for these variables**.**

It should be noted that there are past studies that have shown that Blacks have uniformly worse outcomes than Whites 30, 31, 32, 33, our study is more comprehensive in that we assess nine different ethnic groups and because we adjust for marital, economic, histopathologic, and insurance variables. Our comprehensive analysis allows for a unique finding that Blacks may have better (TP) or similar (Stage IV) outcomes as compared to the White population. Therefore, because the White Hispanic and Black populations present at more advanced stages and have better outcomes, we feel that increased lung cancer screening would be preferentially better in these patients. Such a clear pathway for survival improvement in the White population cannot be ascertained in our population. Unfortunately, SEER does not contain genomic information by race or otherwise. However, at present, genomic information in patients with lung cancer is not prevalent enough (5% or 10% frequency) in ethnic groups other than Whites in the Cancer Genome Atlas (TCGA) 34.

## Conclusion

In summary, our analysis does demonstrate racial disparities do exist in the presentation of the Black and Hispanic populations with lung cancer. Both groups were presented with lower rates of insurance, higher stages, and lower rates of definitive surgery. Blacks had a lower OS/LCSS, but when adjusted for histopathologic, therapeutic, marital status, and economic factors, they had a better OS in the TP than Whites. Disparities in income, marital status, and insurance rather than ethnicity affect OS of patients with lung cancer. Because of their more common presentation with advanced disease, the Black and Hispanic groups may benefit preferentially from screening. Our analyses support the expansion of lung cancer screening to people at higher risk of presenting with advanced stage secondary to limited access to health care due to lower income and lack of insurance, particularly in the Black and Hispanic groups. Specifically, affordable and quality healthcare needs to be provided to these at‐risk populations possibly by education/health literacy and care navigators/coordinators. However, the outcome improvement in the White population may need attention in areas other than just screening.

## Conflict of Interest

None declared.

## Supporting information


**Table S1**. Contains the demographic, histologic, and treatment details in the TP for the nine different ethnic groups.
