# Supplementary material for: Population‐based differences in the outcome and presentation of lung cancer patients based upon racial, histologic, and economic factors in all lung patients and those with metastatic disease
Source: Cancer Med. 2018 Mar 13;7(4):1211–20. doi: 10.1002/cam4.1430 (PMC5911616; doi:10.1002/cam4.1430)

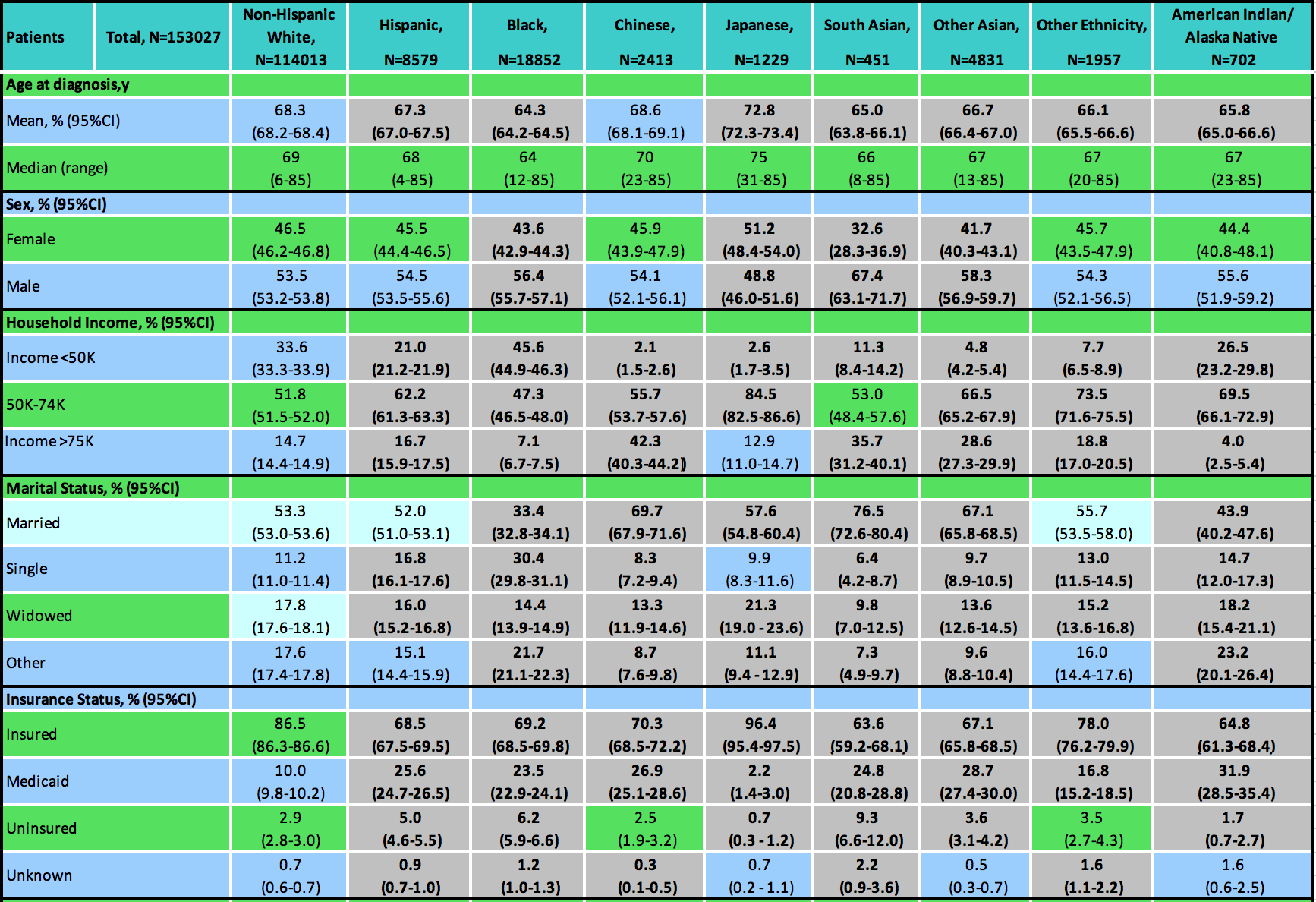
Supplemental Table 1**:** Contains the demographic, histologic, and treatment details in the TP for the nine different ethnic groups. 95% confident intervals are given in parentheses. W is used as reference population. All characteristics differing from the W are in boldprint and brown.


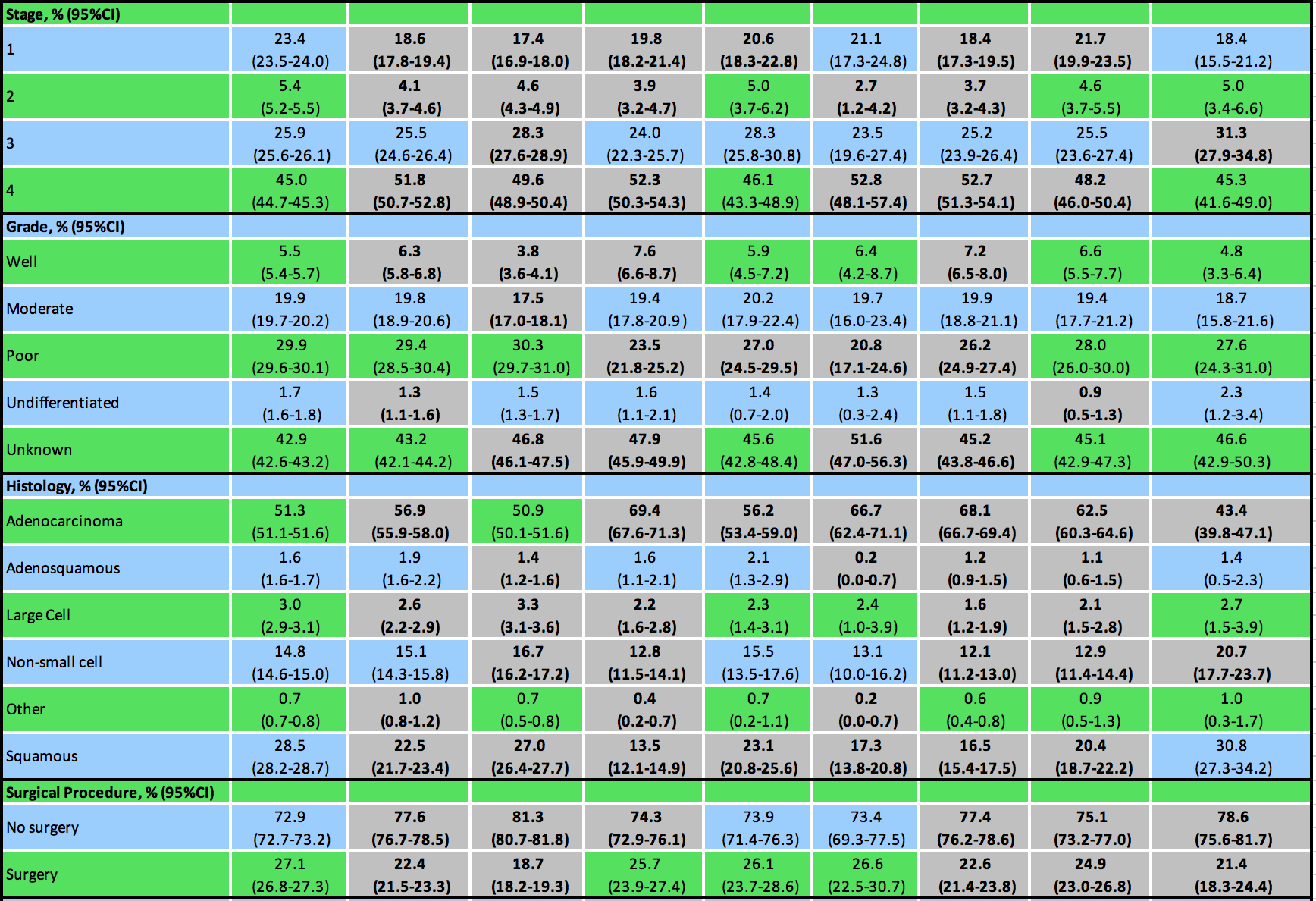


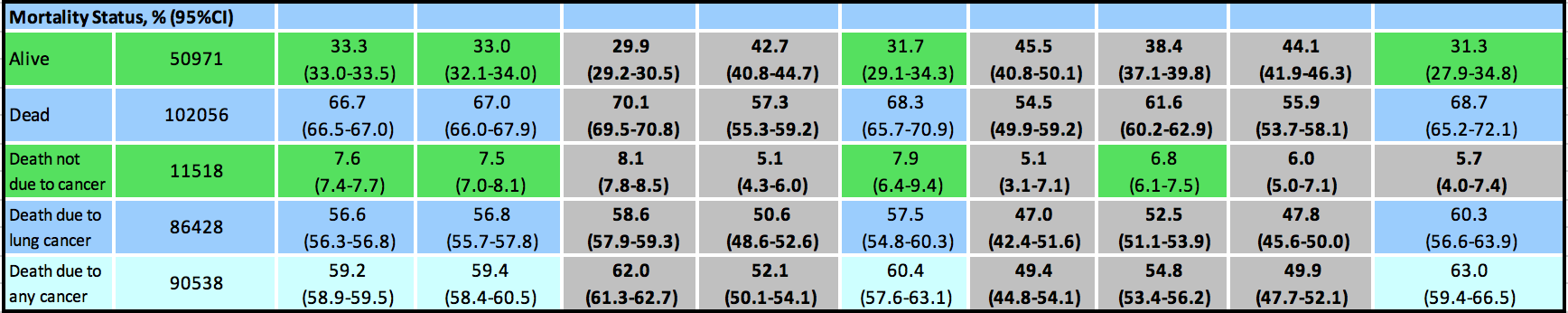

Supplement: Supplementary file 1 — Table S1. Contains the demographic, histologic, and treatment details in the TP for the nine different ethnic groups. [file CAM4-7-1211-s001.docx]
